# In situ analysis of CCR8^+^ regulatory T cells in lung cancer: suppression of GzmB^+^ CD8^+^ T cells and prognostic marker implications

**DOI:** 10.1186/s12885-024-12363-x

**Published:** 2024-05-23

**Authors:** Yoshinori Hayashi, Azumi Ueyama, Soichiro Funaki, Koichi Jinushi, Naoko Higuchi, Hitomi Morihara, Michinari Hirata, Yoji Nagira, Takuro Saito, Atsunari Kawashima, Kota Iwahori, Yasushi Shintani, Hisashi Wada

**Affiliations:** 1 Department of Clinical Research in Tumor Immunology, Graduate School of Medicine, Osaka University, Suita, Osaka 565-0871 Japan; 2 Department of Gastroenterological Surgery, Graduate School of Medicine, Osaka University, Suita, Osaka 565-0871 Japan; 3 Pharmaceutical Research Division, Shionogi & Co., Ltd., -1-1 Futaba-Cho, Toyonaka, Osaka 561-0825 Japan; 4 Department of General Thoracic Surgery, Graduate School of Medicine, Osaka University, Suita, Osaka 565-0871 Japan; 5 Department of Urology, Graduate School of Medicine, Osaka University, Suita, Osaka 565-0871 Japan; 6 Department of Respiratory Medicine and Clinical Immunology, Graduate School of Medicine, Osaka University, Suita, Osaka 565-0871 Japan

**Keywords:** Tumor immunity, Regulatory T cells, CCR8, Cytotoxic T cells, Lung cancer

## Abstract

**Background:**

CCR8-expressing regulatory T cells (Tregs) are selectively localized within tumors and have gained attention as potent suppressors of anti-tumor immunity. This study focused on CCR8^+^ Tregs and their interaction with CD8^+^ T cells in the tumor microenvironment of human lung cancer. We evaluated their spatial distribution impact on CD8^+^ T cell effector function, specifically granzyme B (GzmB) expression, and clinical outcomes.

**Methods:**

A total of 81 patients with lung squamous cell carcinoma (LSCC) who underwent radical surgical resection without preoperative treatment were enrolled. Histological analyses were performed, utilizing an automated image analysis system for double-stained immunohistochemistry assays of CCR8/Foxp3 and GzmB/CD8. We investigated the association of CCR8^+^ Tregs and GzmB^+^ CD8^+^ T cells in tumor tissues and further evaluated the prognostic impact of their distribution profiles.

**Results:**

Histological evaluation using the region of interest (ROI) protocol showed that GzmB expression levels in CD8^+^ T cells were decreased in areas with high infiltration of CCR8^+^ Tregs, suggesting a suppressive effect of CCR8^+^ Tregs on T cell cytotoxicity in the local tumor microenvironment. Analysis of the association with clinical outcomes showed that patients with more CCR8^+^ Tregs and lower GzmB expression, represented by a low GzmB/CCR8 ratio, had worse progression-free survival.

**Conclusions:**

Our data suggest that local CCR8^+^ Treg accumulation is associated with reduced CD8^+^ T cell cytotoxic activity and poor prognosis in LSCC patients, highlighting the biological role and clinical significance of CCR8^+^ Tregs in the tumor microenvironment. The GzmB/CCR8 ratio may be a useful prognostic factor for future clinical applications in LSCC.

**Supplementary Information:**

The online version contains supplementary material available at 10.1186/s12885-024-12363-x.

## Background

Lung cancer is estimated to have 2.2 million new cases and cause 1.8 million deaths each year worldwide, making this disease a major contributor to cancer-related mortality [1, 2]. Among the non-small cell lung cancer subtypes, which constitute approximately 85% of all lung cancer cases, lung adenocarcinoma (LAD) and lung squamous cell carcinoma (LSCC) are the most common [1]. LSCC patients have historically seen scarce benefits from targeted therapies because of the low frequency of driver mutations, such as in *EGFR*, and limited therapeutic efficacy compared with LAD patients [3, 4]. The advent of immune checkpoint inhibitors (ICIs), such as anti-PD-1/PD-L1 or anti-CTLA4 agents, has markedly improved the prognosis of LSCC patients [5, 6]. However, despite these advances, ICIs have limited efficacy and most LSCC patients still do not respond to current immunotherapy methods. This has led to an increased interest in research aiming to understand the tumor immune microenvironment of LSCC.

Regulatory T cells (Tregs) are an immunosuppressive CD4^+^ T cell subset that express the transcription factor Foxp3. They play an important role in maintaining immune tolerance and homeostasis to prevent autoimmune diseases and allergies [7–9]. Tregs also act as immunosuppressors in tumor immunity, potentially inhibiting immune responses against tumor cells and supporting tumor progression [10–12]. CC motif chemokine receptor 8 (CCR8) is a chemokine receptor that has recently been identified using advanced comprehensive transcriptomic analysis and flow cytometry as a novel marker that is selectively expressed on intratumor Tregs [13, 14]. CCR8 is mainly expressed in clonally expanded Tregs activated by tumor-associated antigens, with CCR8^+^ Tregs being an “effector-like” cell population with a stable anti-tumor immunosuppressive function [15]. Our previous study also reported that CCR8^+^ Tregs are involved in the tumor immunosuppressive microenvironment in lung cancer, including LSCC [16]. Furthermore, preclinical studies in several murine tumor models, including lung carcinoma, have demonstrated that depletion of CCR8^+^ Tregs by anti-CCR8 antibody administration resulted in a marked anti-tumor effect [16–22]. Their mechanism of action has been suggested to be via enhancement of CD8^+^ T cell function, as evidenced by the increase in CD8^+^ T cells producing granzyme B (GzmB) and interferon-γ following antibody administration and abolished in vivo efficacy from CD8^+^ T cell depletion [17, 18]. Tumor-infiltrating lymphocytes (TILs) are a vital component of the tumor microenvironment (TME) and are closely related to the progression and prognosis of malignant tumors [23, 24]. CD8^+^ cytotoxic T lymphocytes (CTLs), which can directly kill cancer cells, are a particularly significant T cell subset in anti-tumor immunity. We have previously demonstrated that human CCR8^+^ Tregs from lung cancer TILs or expanded from peripheral blood mononuclear cells have potent inhibitory activity against CTL function in vitro [16]. However, there is no clinical evidence of the impact of CCR8^+^ Tregs on CTL activity in the TME of human cancer patients, which we aimed to evaluate in this study.

Here, we performed histological investigations of human LSCC samples to explore the levels and distribution of CCR8^+^ Tregs and their impact on surrounding CD8^+^ T cells in the local TME. In addition, we analyzed their association with patient clinicopathological features and prognosis to verify their clinical significance.

## Methods

### Patients

We retrospectively identified serial patients who were diagnosed with LSCC and underwent radical resection without preoperative treatment at Osaka University Hospital from March 2010 to May 2017. Excluded individuals included those who underwent palliative or non-curative resection and those with multiple concurrent cancers. A total of 81 patients were included in the study. Medical data of the patients, such as clinicopathological characteristics, surgical findings, and clinical course, were collected retrospectively using the clinical database and pathological examination reports of the institute. Tumor staging was performed according to the eighth edition of the UICC TNM classification system [25]. Nine of 81 patients received the following postoperative treatments: tegafur-uracil (1 patient), carboplatin plus paclitaxel (5 patients) or nab-paclitaxel (2 patients) or S-1 (1 patient). All patients were monitored for recurrence and death or survival to the last follow-up (at least 5 years) for recurrence-free cases. This study was conducted in accordance with the principles of the Declaration of Helsinki and was approved by the Institutional Review Board of Osaka University Hospital (approval number: 13266). All participants provided informed consent using the opt-out methodology from the retrospective design of the study.

### Double staining for immunohistochemistry (IHC) analysis

Formalin-fixed paraffin-embedded (FFPE) surgical specimens from each patient were collected and prepared. Three serial 4 µm thin sections were cut for hematoxylin and eosin (H&E) staining and double IHC staining for CCR8/Foxp3 and GzmB/CD8, respectively. The sections were deparaffinized using xylene, subjected to a stepwise ethanol dilution series for hydration, and then immersed in an EDTA-based antigen retrieval buffer (pH 9.0), followed by incubation in a pressure cooker at 110 °C for 15 min. The sections were washed in distilled water, incubated for 10 min in 3% hydrogen peroxide (H_2_O_2_) to block endogenous peroxidase activity, washed three times in 0.05% Tween-20/TBS, and then incubated for 20 min in a 3% bovine serum albumin or 5% goat serum solution/PBS for non-specific antigen reaction blocking. The antibodies were diluted in 1% bovine serum albumin/PBS. The sections were incubated 2 h at room temperature with primary antibodies (for CCR8 and CD8). After washing three times, detection was performed using a polymer reagent (Histofine Simple Stain MAX PO, Nichirei Bioscience Inc., Tokyo, Japan) and 3’-3-diamnobenzidine (DAB) substrate according to the manufacturer's protocol. Subsequently, antigen retrieval was performed again, followed by 3% H_2_O_2_ treatment and blocking. The samples were then incubated 2 h at room temperature with other primary antibodies (for Foxp3 and GzmB). They were washed three times, followed by detection with a polymer reagent and Vina Green Chromogen kit (BRR807AH, Biocare Medical LLC, Pacheco, CA, USA). The sections were counterstained with hematoxylin, washed, dehydrated with ethanol, and then mounted. The primary antibodies used were as follows: CCR8 (433H, mouse monoclonal, BD Biosciences, Franklin Lakes, NJ, USA, 1:40 dilution), CD8 (L26, mouse monoclonal, Nichirei Bioscience, Tokyo, Japan, ready to use), Foxp3 (236A/E, mouse monoclonal, Abcam, Cambridge, UK, 1:100 dilution), and GzmB (D6E9W, rabbit monoclonal, Cell Signaling Technology, Danvers, MA, USA, 1:50 dilution).

### Analysis of IHC staining images

The double-stained glass slides were scanned and merged into digital slide images at 40 × magnification using a microscope slide scanner system (VENTANA iScan HT, Roche-Ventana Medical Systems Inc., Tucson, AZ, USA). Images were imported into a pathological image analysis software (HALO version 3.5, Indica Labs, Albuquerque, NM, USA), then all sequential steps for automated analysis, including annotation, training, and analysis, were performed [26]. The analysis step was carried out using the auto-cell counting algorithm generated in the training step. Tumor lesions in each slide were discriminated by observation of H&E-stained images by an experienced pathologist. The following measurements within the tumor lesions were obtained according to the Whole Tumor Area (WTA) protocol or Region of Interest (ROI) protocol:

$$\begin{array}{c}\left[{\mathrm{CCR}8}^+\;\mathrm{Treg}\right]\;={\mathrm{CCR}8}^+{\mathrm{Foxp}3}^+\;\mathrm{cell}\;\mathrm{counts}\;\mathrm{per}\;\mathrm{area}.\\\left[\mathrm{Total}\;{\mathrm{CD}8}^+\;\mathrm T\right]\;={\mathrm{CD}8}^+\mathrm{cell}\;\mathrm{count}\;\mathrm{per}\;\mathrm{area}.\\\left[\mathrm{GzmB}^+\;{\mathrm{CD}8}^+\;\mathrm T\right]\;=\mathrm{GzmB}^+{\mathrm{CD}8}^+\;\mathrm{cell}\;\mathrm{count}\;\mathrm{per}\;\mathrm{area}.\\\left[\%\mathrm{GzmB}^+\;\mathrm{in}\;{\mathrm{CD}8}^+\;\mathrm T\right]\;={\mathrm{percentage}\;\mathrm{of}\;\mathrm{GzmB}}^+\;\mathrm{cells}\;\mathrm{out}\;\mathrm{of}\;{\mathrm{CD}8}^+\;\mathrm{cells}.\end{array}$$

In the WTA protocol, the overall tumor area of each tissue section was assessed. In the ROI protocol, cell density heat maps were drawn from CCR8/Foxp3-stained images using the spatial analysis mode of HALO. Multiple fields (360 × 270 µm in size) with high positive cell counts were obtained and the top five fields were selected as representative “Hot Spots” for analysis. Five fields with few CCR8^+^ Tregs were also selected as representative “Cold Spots” for analysis, except for fields with less than 10 positive cells for both CD8 and Foxp3 staining. CCR8/Foxp3-stained and CD8/GzmB-stained images of the matched fields were analyzed.

For the association analysis of clinical features and prognosis, measurements obtained from overall tissue analysis by the WTA protocol or the average of the measurements obtained from five Hot Spots per case by the ROI protocol were used as representative values for each patient. The [GzmB/CCR8 ratio] is defined as [%GzmB^+^ in CD8^+^ T] divided by [CCR8^+^ Treg].

### Statistical analysis

Statistical analysis and data description were performed using JMP Pro software version 16.2.0 (SAS Institute Inc., Cary, NC, USA) and R version 4.3.1 (R Foundation for Statistical Computing, Vienna, Austria). A linear regression model was used for correlation analysis. Comparisons between two groups were analyzed using the Mann–Whitney U test for continuous variables and Fisher’s exact test for categorical variables. Progression-free survival (PFS) was estimated using the Kaplan–Meier method and compared using the log-rank test. Hazard ratios (HRs) with a 95% confidence interval (CI) were calculated using the Cox proportional hazards model. A *P*-value < 0.05 was considered statistically significant.

## Results

### Whole tumor tissue assessment of CCR8^+^ Tregs and GzmB^+^CD8^+^ T cells

**Fig. 1:**
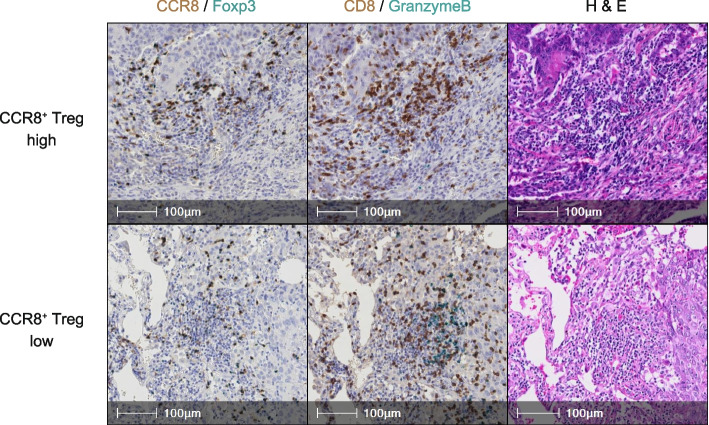
Representative images of double-stained immunohistochemistry assays. The left panel shows CCR8 (brown) and Foxp3 (blue) staining, the middle panel shows CD8 (brown) and GzmB (blue) staining, and the right panel shows hematoxylin and eosin (H&E)-stained images. The three top images are from the CCR8^+^ Treg high case and the three bottom images are from the CCR8^+^ Treg low case

**Fig. 2:**
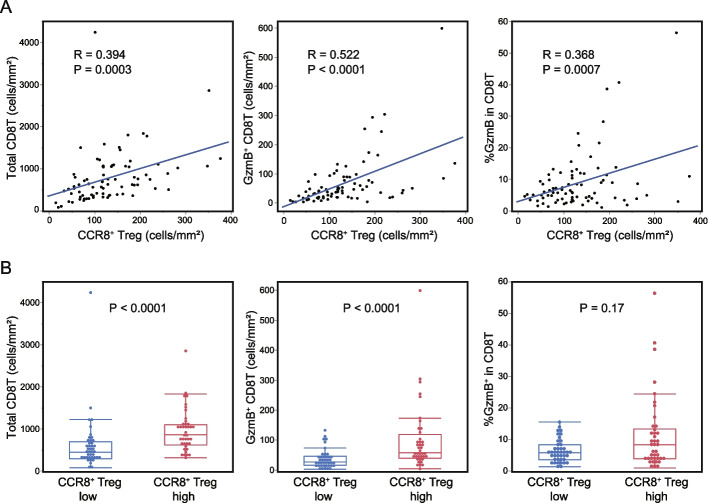
Association of CCR8^+^ Tregs with CD8^+^ T cell parameters by the whole tumor area (WTA) analysis protocol. The overall tissue area of CCR8/Foxp3 and CD8/GzmB double-stained tissue sections from 81 lung squamous cell carcinoma (LSCC) patients were analyzed. **A** Correlation plots with the linear regression model are displayed. **B** All 81 patients were divided into high and low groups relative to the [CCR8^+^ Treg (mm^2^)] median value. The two groups were compared by the Mann–Whitney U test

Serial tissue sections from 81 LSCC patients were double-stained for CCR8/Foxp3 and GzmB/CD8. Representative images of the IHC staining results for two typical cases with high and low CCR8^+^ Treg infiltration are shown in Fig. 1. First, we examined the relationship between CCR8^+^ Treg infiltration and CD8^+^ T cell infiltration or GzmB expression by using WTA analysis. The results showed that [CCR8^+^ Treg (cells/mm^2^)] had a weak positive correlation with [Total CD8^+^ T (cells/mm^2^)], [GzmB^+^ CD8^+^ T (cells/mm^2^)], and [%GzmB^+^ in CD8^+^ T] (Fig. 2A). When patients were divided into high and low groups relative to the [CCR8^+^ Treg (cells/mm^2^)] median value, the [CCR8^+^ Treg]-high group had higher infiltration of [Total CD8^+^ T (cells/mm^2^)] and [GzmB^+^ CD8^+^ T (cells/mm^2^)], while no significant association was observed with [%GzmB^+^ in CD8^+^ T] (Fig. 2B).

### Analysis of CCR8^+^ Tregs and GzmB^+^CD8^+^ T cells in ROIs with high immune cell infiltration

**Fig. 3:**
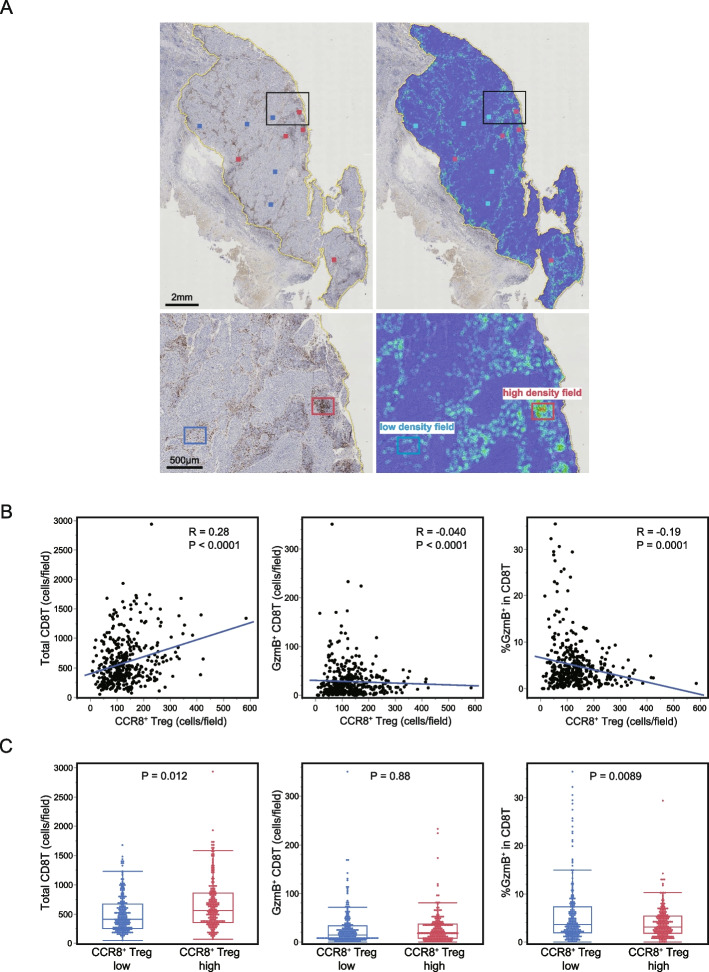
Association of CCR8^+^ Tregs with CD8^+^ T cell parameters by the region of interest (ROI) analysis protocol. **A** Representative whole tissue scan image of CCR8/Foxp3-stained sections. The left panel shows a double-stained immunohistochemistry image and the right panel shows a density heat map of positive cells. Positive cells exhibit heterogeneous distribution, with areas of high density (red box) and low density (blue box). **B** Five Hot Spots with high CCR8^+^ Treg infiltration were selected per case for 81 lung squamous cell carcinoma (LSCC) patients and the CCR8/Foxp3 and GzmB/CD8 stained images of the matched fields were analyzed. The correlation plots with the linear regression model of the data from 405 fields are presented. **C** Data from all 405 fields were divided into high and low groups relative to the [CCR8^+^ Treg (cells/filed)] median value. The two groups were compared by the Mann–Whitney U test

The overall tumor assessment with the WTA protocol showed a weak positive correlation between CCR8^+^ Treg infiltration and GzmB expression in CD8^+^ T cells. However, the stained images represented in Fig. 1 suggest that GzmB expression levels in CD8^+^ T cells appear to be lower in high CCR8^+^ Treg cases compared with in low CCR8^+^ Treg cases. Observation of the whole tissue scan image, shown in Fig. 3A, revealed that the immune cell distribution is heterogeneous, with areas of high-density, low-density, and no lymphocytes. Therefore, to investigate the effects of local interactions between CCR8^+^ Tregs and CD8^+^ T cells, we focused our analysis on specific ROIs where the CCR8^+^ Tregs are highly accumulated.

Using the density heat map generated by spatial analysis, Hot Spots with high CCR8^+^ Treg infiltration were selected for five fields in each case (Fig. 3A) and GzmB/CD8-stained images of the matched fields were analyzed. This ROI analysis showed that [CCR8^+^ Treg (cells/field)] positively correlated with [Total CD8^+^ T (cells/field)], but negatively correlated with [GzmB^+^ CD8^+^ T (cells/ field)] and [%GzmB^+^ in CD8^+^ T] (Fig. 3B). In a two-group comparison, [Total CD8^+^ T (cells/ field)] was significantly higher, whereas [%GzmB^+^ in CD8^+^ T] was significantly lower, in the high [CCR8^+^ Treg (cells/ field)] areas than in the low areas (Fig. 3C).

In contrast, the analysis of total Foxp3^+^ Tregs showed that [Treg (cells/field)] had a positive correlation with [Total CD8^+^ T (cells/field)] and [GzmB^+^ CD8^+^ T (cells/field)], and a weaker negative correlation with [%GzmB^+^ in CD8^+^ T] than [CCR8^+^ Treg (cells/field)] (Supplementary Figure S1A). Furthermore, there was no significant difference in [%GzmB^+^ in CD8^+^ T] between areas with high and low [Total Treg (cells/field)] (Supplementary Figure S1B).

To further evaluate the impact of CCR8^+^ Treg local accumulation on neighboring CD8^+^ T cells in the TME, we analyzed GzmB^+^ CD8^+^ T cells in areas of high and low CCR8^+^ Treg infiltration within the same case. In addition to Hot Spots, low-density fields of CCR8^+^ Tregs (excluding areas with no lymphocytes) were selected as Cold Spots for five fields in each case. Scatter plots of [CCR8^+^ Treg (cells/field)] and each CD8^+^ T cell parameter in each case are shown in Supplementary Figure S2. A correlation analysis using the Hot and Cold Spot data from 40 cases with high CCR8^+^ Treg infiltration showed that [CCR8^+^ Treg (cells/field)] was positively correlated with [Total CD8^+^ T (cells/field)], but negatively correlated with [%GzmB^+^ in CD8^+^ T] (Supplementary Figure S3A). Furthermore, [%GzmB^+^ in CD8^+^ T] in Hot Spots was significantly decreased compared with in Cold Spots (Supplementary Figure S3B). These results suggest that CD8^+^ T cell cytotoxicity may be suppressed in areas of the TME with CCR8^+^ Treg accumulation.

### Patients with more CCR8^+^ Tregs and lower GzmB expression levels had a poorer prognosis

**Fig. 4:**
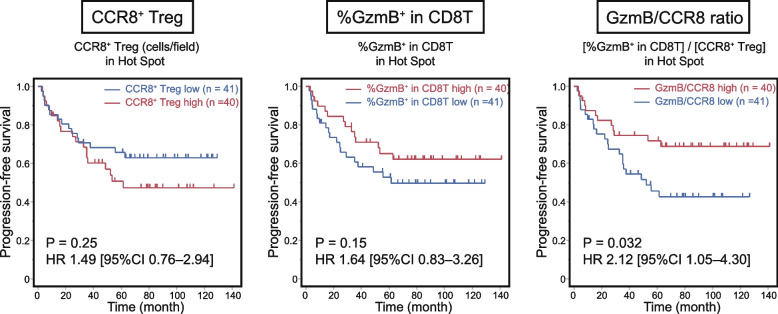
Prognostic impact of CCR8^+^ Tregs and GzmB^+^ CD8^+^ T cells in the tumor microenvironment (TME). Using the average measurements obtained from five Hot Spots per case with the region of interest (ROI) analysis, 81 lung squamous cell carcinoma (LSCC) patients were divided into high and low groups relative to the median value of each measurement. The Kaplan–Meier survival curves for progression-free survival (PFS) are presented. The GzmB/CCR8 ratio is defined as [%GzmB^+^ in CD8^+^ T] divided by [CCR8^+^ Treg (cells/field)]. Group comparisons were conducted using the log-rank test. Hazard ratios (HRs) with the 95% confidence interval (CI) were calculated using the Cox proportional hazards model

Finally, we investigated the association between the immunosuppressive profile indicated by our histological analysis and clinical outcomes, specifically PFS. All patients were divided into two groups relative to the median value of [CCR8^+^ Treg (cells/field)] or [%GzmB^+^ in CD8^+^ T] at Hot Spots from the ROI protocol. As shown in Fig. 4, the [CCR8^+^ Treg (cells/field)]-high group had worse PFS than the low group (3-year PFS 60.2% vs. 70.7%, respectively, *P* = 0.25) and the [%GzmB^+^ in CD8^+^ T]-low group had worse PFS than the high group (3-year PFS 59.7% vs. 71.7%, respectively, *P* = 0.15), but neither result was statistically significant. We then divided the patients based on the GzmB/CCR8 ratio, representing local CCR8^+^ Treg accumulation and reduced CD8^+^ T cell cytotoxicity. The data suggested that the low patient group had significantly worse PFS than the high group (3-year PFS 57.1% vs. 74.5%, respectively, *P* = 0.032). A comparable trend was observed with the WTA protocol, but there were no significant differences in any of the indices (Supplementary Figure S4). Furthermore, similar analyses were performed for the CD8/Foxp3 ratio, GzmB/Foxp3 ratio, and CD8/CCR8 ratio, none of which showed significant differences in PFS (Supplementary Figure S5). When comparing clinicopathological characteristics, the low GzmB/CCR8 ratio group from the ROI protocol had significantly higher rates of lymph node metastasis (34.1% vs. 12.5%, respectively, *P* = 0.035) and pleural invasion (22.0% vs. 5.0%, respectively, *P* = 0.048) (Table 1). Multivariate analyses for PFS demonstrated that the GzmB/CCR8 ratio from the ROI protocol was an independent prognostic factor (HR = 2.13, 95% CI = 1.05–4.32, *P* = 0.036), along with pStage (HR = 2.08, 95% CI = 1.02–4.23, *P* = 0.044) (Table 2).

**Table 1:** Clinicopathological characteristics of lung squamous cell carcinoma (LSCC) patients according to CCR8^+^ Treg, %GzmB^+^ in CD8^+^ T, and the GzmB/CCR8 ratio

Variables	CCR8^+^ Treg high (*n* = 40)	CCR8^+^ Treg low (*n* = 41)	*P*-value	%GzmB^+^ in CD8^+^ T high (*n* = 40)	%GzmB^+^ in CD8^+^ T low (*n* = 41)	*P*-value	GzmB/CCR8 ratio high (*n* = 40)	GzmB/CCR8 ratio low (*n* = 41)	*P*-value
Age in years, median (range)	71.5 (42—85)	72 (38—85)	0.94	71 (42—85)	74 (38—83)	0.12	71.5 (38–85)	72 (58–83)	0.65
Sex			0.15			0.15			0.48
Female	6 (15.0)	2 (4.9)		6 (15.0)	2 (4.9)		5 (12.5)	3 (7.3)	
Male	34 (85.0)	39 (95.1)		34 (85.0)	39 (95.1)		35 (87.5)	38 (92.7)	
Smoking status			0.62			0.62			0.62
Never	2 (5.0)	1 (2.4)		2 (5.0)	1 (2.44)		2 (5.0)	1 (2.4)	
Current/former	38 (95.0)	40 (97.6)		38 (95.0)	40 (97.6)		38 (95.0)	40 (97.6)	
Brinkmann index, median (range)	1120 (0–3900)	1032 (0–3430)	0.50	1036 (0–3900)	1060 (0–3500)	0.94	1000 (0–3900)	1200 (0–3500)	0.22
Tumor laterality			0.82			1.00			0.82
Left	19 (47.5)	18 (43.9)		18 (45.0)	19 (46.3)		19 (47.5)	18 (43.9)	
Right	21 (52.5)	23 (56.1)		22 (55.0)	22 (53.7)		21 (52.5)	23 (56.1)	
Tumor location			0.66			1.00			0.66
Upper lobe/Middle lobe	22 (55.0)	25 (61.0)		23 (57.5)	24 (58.5)		25 (61.0)	22 (55.0)	
Lower lobe	18 (45.0)	16 (39.0)		17 (42.5)	17 (41.5)		16 (39.0)	18 (45.0)	
Surgical procedure			1.00			0.52			0.76
Wedge resection/segmentectomy	5 (12.5)	6 (14.6)		4 (10.0)	7 (17.1)		6 (15.0)	5 (12.2)	
Lobectomy/pneumonectomy	35 (87.5)	35 (85.4)		36 (90.0)	34 (82.9)		34 (85.0)	36 (87.8)	
Postoperative treatment (chemotherapy)			0.31			0.74			0.48
Yes	6 (15.0)	3 (7.3)		5 (12.5)	4 (9.8)		3 (7.5)	6 (14.6)	
No	34 (85.0)	38 (92.7)		35 (87.5)	37 (90.2)		37 (92.5)	35 (85.4)	
Histological differentiation (SCC)			0.26			0.15			0.80
Well/moderate	38 (95.0)	35 (85.4)		34 (85.0)	39 (95.1)		31 (77.5)	30 (73.2)	
Poor	2 (5.0)	6 (14.6)		6 (15.0)	2 (4.9)		9 (22.5)	11 (26.8)	
pT			0.59			0.59			1.00
T1—2	33 (82.5)	31 (75.6)		33 (82.5)	31 (75.6)		32 (80.0)	35 (78.1)	
T3—4	7 (17.5)	10 (24.4)		7 (17.5)	10 (24.4)		8 (20.0)	10 (22.0)	
pN			**0.019**			0.30			**0.035**
N0	26 (65.0)	36 (87.8)		33 (82.5)	29 (70.7)		35 (87.5)	27 (65.9)	
N1—2	14 (35.0)	5 (12.2)		7 (17.5)	12 (29.3)		5 (12.5)	14 (34.1)	
pStage			0.27			0.37			0.37
Stage I	20 (50.0)	26 (63.4)		25 (62.5)	21 (51.2)		25 (62.5)	21 (51.2)	
Stage II/III	20 (50.0)	15 (36.6)		15 (37.5)	20 (48.8)		15 (37.5)	20 (48.8)	
Lymphatic invasion			0.35			**0.048**			0.19
Absent	33 (82.5)	37 (90.2)		38 (95.0)	32 (78.1)		37 (92.5)	33 (80.5)	
Present	7 (17.5)	4 (9.8)		2 (5.0)	9 (22.0)		3 (7.5)	8 (19.5)	
Vascular invasion			0.19			0.088			0.088
Absent	33 (82.5)	38 (92.7)		38 (95.0)	33 (80.5)		38 (95.0)	33 (80.5)	
Present	7 (17.5)	3 (7.3)		2 (5.0)	8 (19.5)		2 (5.0)	8 (19.5)	
Pleural invasion			0.35			**0.0070**			**0.048**
Absent	33 (82.5)	37 (90.2)		39 (97.5)	31 (75.6)		38 (95.0)	32 (78.0)	
Present	7 (17.5)	4 (9.8)		1 (2.5)	10 (24.4)		2 (5.0)	9 (22.0)	

Data are presented as n (%) unless noted otherwise

Bolded *P*-values indicate statistical significance (*P* < 0.05)

*Abbreviations*: *CCR8* CC motif chemokine receptor 8, *Treg* regulatory T cell, *GzmB* Granzyme B

**Table 2:** Univariate and multivariate analyses of progression-free survival in lung squamous cell carcinoma (LSCC) patients

Variables	Univariate analysis		Multivariate analysis	
	HR (95% CI)	*P*-value	HR (95% CI)	*P*-value
Age		0.13		
< 72 years	1 [Reference]			
≥ 72 years	1.69 (0.85–3.35)			
Sex		0.15		
Female	1 [Reference]			
Male	4.33 (0.59–31.71)			
Smoking status		0.86		
Never/former	1 [Reference]			
Current	1.19 (0.16–8.73)			
Brinkmann index		0.18		
< 1040	1 [Reference]			
≥ 1040	1.61 (0.81–3.19)			
Tumor laterality		0.58		
Left	1 [Reference]			
Right	1.21 (0.62–2.39)			
Tumor Location		0.016		0.086
Ul/Ml	1 [Reference]		1 [Reference]	
Ll	2.32 (1.17–4.60)		1.87 (0.91–3.82)	
Surgical procedure		0.91		
Wedge resection/segmentectomy	1.06 (0.41–2.74)			
Lobectomy/pneumonectomy	1 [Reference]			
Postoperative treatment		0.97		
Yes	1.02 (0.36–2.91)			
No	1 [Reference]			
Histological differentiation (SCC)		0.64		
Well/moderate	1 [Reference]			
Poor	1.30 (0.46–3.70)			
pStage		0.0056		**0.044**
I	1 [Reference]		1 [Reference]	
II, III	2.62 (1.32–5.17)		2.08 (1.02–4.23)	
GzmB/CCR8 ratio		0.037		**0.036**
High	1 [Reference]		1 [Reference]	
Low	2.12 (1.05–4.30)		2.13 (1.05–4.32)	

*Abbreviations*: *HR* hazard ratio, *CI* confidence interval, *Ul* upper lobe, *Ml* middle lobe, *Ll* lower lobe, *SCC* squamous cell carcinoma, *GzmB* Granzyme B, *CCR8* CC motif chemokine receptor 8

## Discussion

This study focused on the interactions between CCR8^+^ Tregs and CD8^+^ T cells in the LSCC TME, evaluating the impact of their spatial distribution on anti-tumor immunity by histological analysis. As a measure of CD8^+^ T cell function, we analyzed a potent cytotoxic molecule GzmB, which is present in cytotoxic granules and released upon contact with cancer cells to kill them. We found that local accumulation of CCR8^+^ Tregs within tumors was associated with reduced GzmB expression levels in closely infiltrating CD8^+^ T cells, as well as that the GzmB/CCR8 ratio was an independent prognostic factor in LSCC.

CCR8^+^ Tregs have recently attracted attention as immunosuppressive cells that are highly concentrated in malignant tumor tissues compared with in peripheral blood and normal tissues in both humans and mice [19–22]. Using flow cytometry and public mRNA database analysis, our previous study showed high CCR8^+^ Treg infiltration within tumors that was associated with poor prognosis in lung cancer patients [16]. Ex vivo culture studies suggested that CCR8^+^ Tregs can suppress the cytotoxic function of CD8^+^ T cells [16]. Mouse studies have also corroborated that CCR8^+^ Tregs can suppress anti-tumor immunity via regulation of CD8^+^ T cell function [17, 18]. However, to our knowledge, our data in this study provide the first evidence suggesting that CCR8^+^ Tregs can suppress CD8^+^ T cell cytotoxic activity in vivo within the TME of human cancer patients. Notably, the results were more robust for the analysis of CCR8^+^ Tregs than for the analysis of total Tregs, highlighting the importance of CCR8^+^ Tregs for the suppressive effect.

In this study, tumor-infiltrating immune cells were evaluated using two protocols: the WTA protocol and ROI protocol. However, the results of the two protocols were not consistent. An overall assessment using the WTA protocol showed that high CCR8^+^ Treg infiltration was positively correlated with both high GzmB^+^CD8^+^ T cell infiltration and high GzmB expression in CD8^+^ T cells, with no findings suggesting immunosuppression by CCR8^+^ Tregs. This would indicate that patients with a high number of TILs have both functional and suppressive T cell infiltration and activation throughout the tumor tissue, which is consistent with several previous reports [27–29]. However, full-frame imaging of tumor sections showed heterogeneous accumulation of TILs. In general, immune cell infiltration within tumors is heterogeneously distributed, making spatial analysis of TILs in the local microenvironment important for fully assessing anti-tumor immune activity [30, 31]. Several previous reports have highlighted the importance of examining local TIL infiltration, focusing on the Hot Spots of high infiltration, tumor stroma, and tumor periphery. These serve as biomarkers that are relevant to prognosis and therapeutic efficacy in various types of cancer [32–36]. Therefore, we performed an ROI analysis to investigate the effect of CCR8^+^ Tregs in the local TME. Even in the ROI analysis in Hot Spot, the number of CCR8^+^ Tregs and CD8^+^ T cells were positively correlated. Our previous studies have shown that CCR8^+^ Tregs have an activated chemokine-related pathway and high expression of various chemokines and chemokine receptors [16]. Therefore, CCR8^+^ Tregs may be recruited around effector cells and also recruit effector cells to their surroundings, forming Hot Spots where immune cells accumulate. In contrast, unlike the results by the WTA analysis, our ROI analysis in Hot Spots revealed a negative correlation between CCR8^+^ Treg accumulation and GzmB expression patterns in CD8^+^ T cells, supporting the possibility of in situ CTL suppression.

Many studies have reported the clinical utility of TIL evaluation. The most well-known approach is the Immunoscore, which reflects the level of CD3^+^ and CD8^+^ T lymphocyte infiltration and has been established as a powerful prognostic indicator in colorectal cancer [37]. In lung cancer, in situ TIL assessment has also been recognized as an important prognostic tool and similar approaches focusing on CD3^+^ or CD8^+^ T cell infiltration have been investigated [38–40]. Conversely, the high Foxp3/CD3 or Foxp3/CD8 ratio in tumor tissues has been reported as a poor prognostic factor [41–43], suggesting that the balance between CD8^+^ T cells and Tregs is important in tumor immunity. Here, we propose that the GzmB/CCR8 ratio, which combines CCR8^+^ Treg accumulation and GzmB expression in CD8^+^ T cells, is a more advanced prognostic marker. Importantly, the GzmB/CCR8 ratio was more strongly associated with prognosis in LSCC patients than the CD8/Foxp3 ratio, GzmB/Foxp3 ratio, or CD8/CCR8 ratio. This indicator is expected to sensitively reflect the activity of anti-tumor immunity from the perspective of both immune promoters and suppressors, with the potential to provide reasonable and good prognostic prediction. Moreover, our results provide evidence to support the potential of CCR8-targeted therapies which are currently in development. These therapies are expected to be more effective in patients with the low GzmB/CCR8 ratio, an index of local CD8^+^ T cell suppression by CCR8^+^ Tregs.

This study has several limitations. First, it is an observational study with a limited number of cases from a single institution. Further prospective verification with a larger sample size is necessary in the future. Second, our analysis did not separate the stromal and intra-tumoral areas despite there being a potential relationship between the distribution of TILs and histological structure, as discussed above. While the automated ROI acquisition method used here is valid, we did not define the tumor area within the ROI. Third, the study did not fully investigate the mechanism by which CCR8^+^ Tregs can suppress GzmB expression in CD8^+^ T cells. In our previous study, we experimentally demonstrated that the CCR8^+^ Treg-mediated inhibition of CD8^+^ T cell function can be canceled by blocking major histocompatibility complex (MHC) molecules [16], suggesting that the suppressive effect of CCR8^+^ Tregs is mediated by antigen-presenting cells (APCs). However, we did not assess APCs or MHC molecules in this study. This will be explored in future research.

## Conclusions

Our histological study illustrated that the accumulation of CCR8^+^ Tregs in the TME may lead to reduced cytotoxic function of the adjacent CD8^+^ T cells and poor prognosis in LSCC patients. This highlights the biological importance and clinical relevance of CCR8^+^ Tregs in anti-tumor immunity. The GzmB/CCR8 ratio is a potential prognostic predictor for LSCC and may benefit future clinical applications.

### Supplementary Information

Supplementary Material 1.

Supplementary Material 2.

Supplementary Material 3.

Supplementary Material 4.

Supplementary Material 5.

## Data Availability

The datasets used and/or analysed during the current study are available from the corresponding author on reasonable request.

## Abbreviations

APC: Antigen-presenting cell
CCR8: CC motif chemokine receptor 8
CI: Confidential interval
CTL: Cytotoxic T lymphocyte
DAB: 3’-3-Diamnobenzidine
FFPE: Formalin-fixed paraffin embedded
FoxP3: Forkhead box protein 3
GzmB: Granzyme B
H_2_O_2_: Hydrogen peroxide
HR: Hazard Ratio
H&E: Hematoxylin and eosin
ICI: Immune checkpoint inhibitors
IHC: Immunohistochemistry
LAD: Lung adenocarcinoma
LSCC: Lung squamous cell carcinoma
MHC: Major histocompatibility complex
PD-L1: Programmed death ligand 1
PD-1: Programmed cell death 1
PFS: Progression-free survival
ROI: Regions of Interest
Treg: Regulatory T cell
TIL: Tumor-infiltrating lymphocyte
TME: Tumor microenvironment
WTA: Whole tumor area
