# Supplementary material for: In situ analysis of CCR8+ regulatory T cells in lung cancer: suppression of GzmB+ CD8+ T cells and prognostic marker implications
Source: BMC Cancer. 2024 May 23;24:627. doi: 10.1186/s12885-024-12363-x (PMC11112935; doi:10.1186/s12885-024-12363-x)
Supplement: Supplementary file 1 — Supplementary Material 1. [file 12885_2024_12363_MOESM1_ESM.pdf]

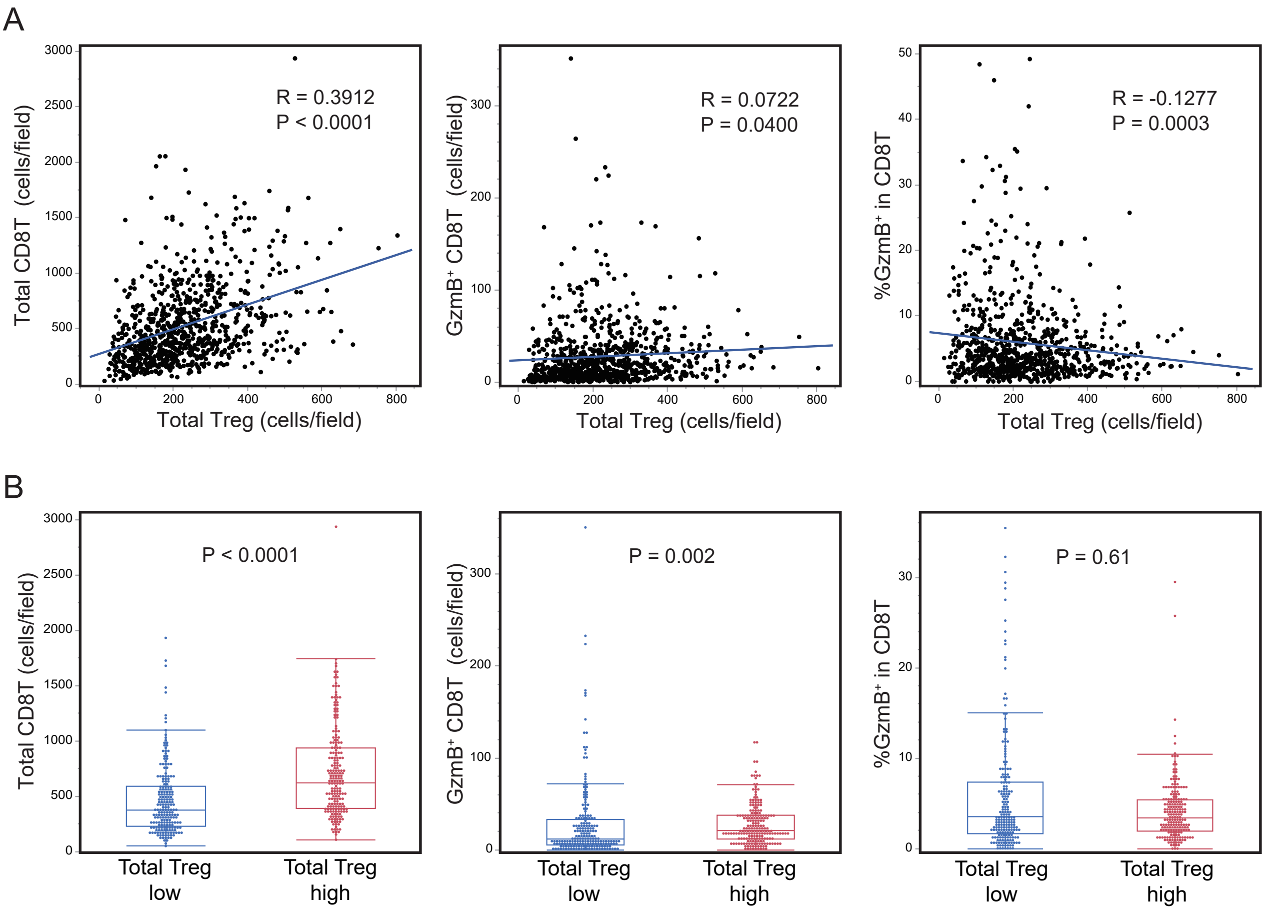

Supplementary Figure S1. Association of total Foxp3^{+} Tregs with CD8^{+} T cell parameters by the region of interest (ROI) analysis protocol. **A.** Five Hot Spots with high Treg infiltration were selected per case for 81 patients. The correlation plots with linear regression model of the data from 405 fields are presented. **B.** The data from all 405 fields were divided into high and low groups relative to the [Total Treg (cells/filed)] median value. The two groups were compared by the Mann-Whitney U test.
