# Supplementary material for: In situ analysis of CCR8+ regulatory T cells in lung cancer: suppression of GzmB+ CD8+ T cells and prognostic marker implications
Source: BMC Cancer. 2024 May 23;24:627. doi: 10.1186/s12885-024-12363-x (PMC11112935; doi:10.1186/s12885-024-12363-x)
Supplement: Supplementary file 2 — Supplementary Material 2. [file 12885_2024_12363_MOESM2_ESM.pdf]

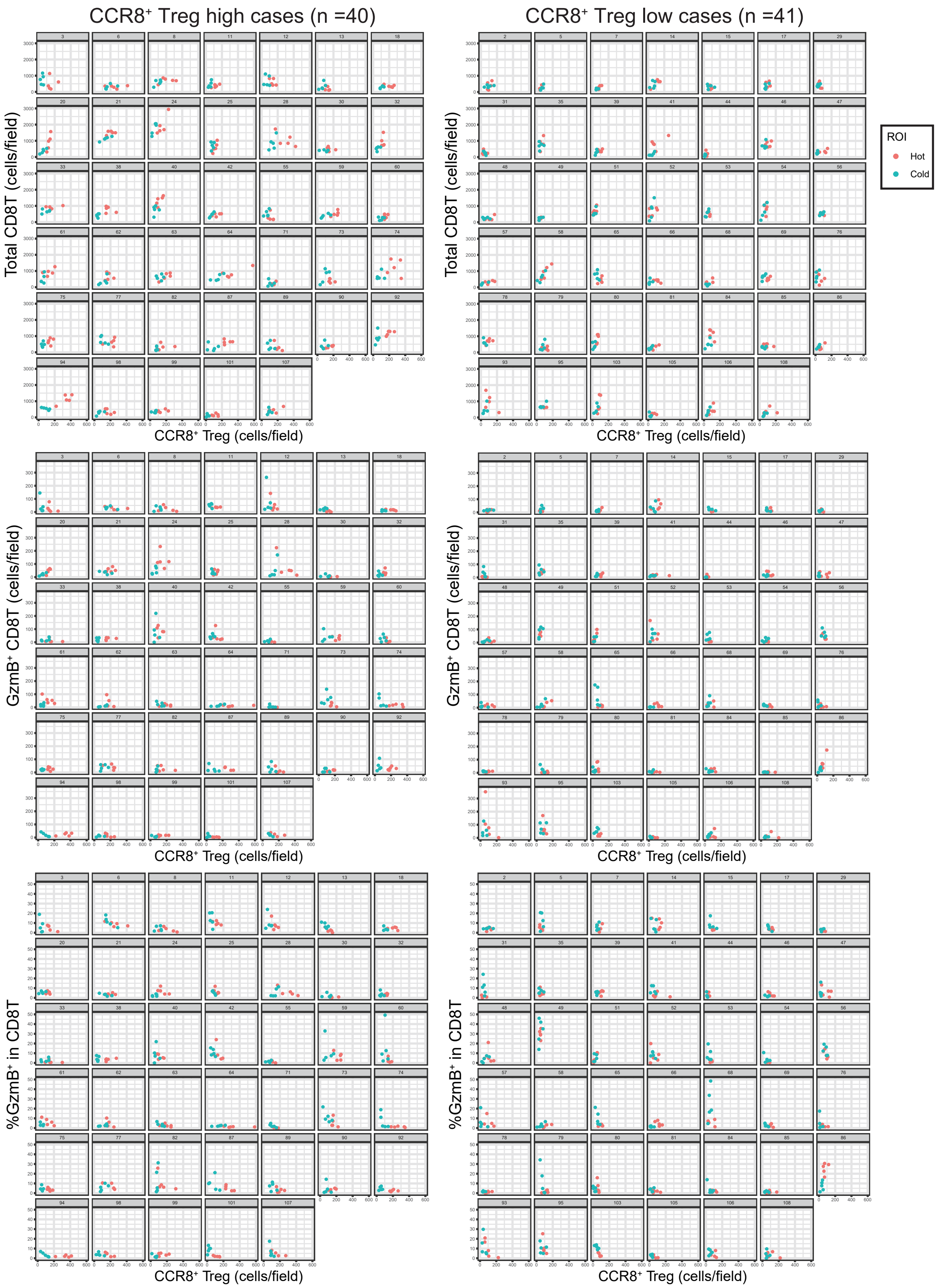

Supplementary Figure S2. Association of CCR8^{+} Tregs with CD8^{+} T cell parameters in each case. Five Hot Spots and five Cold Spots were selected per case for 81 lung squamous cell carcinoma (LSCC) patients and the CCR8/Foxp3 and GzmB/CD8 stained images of the matched fields were analyzed. The left panels show the scatter plots of patients with high CCR8^{+} Treg infiltration in the Hot Spots and the right panels show the scatter plots of patients with low CCR8^{+} Treg infiltration in the Hot Spots.
