# Supplementary material for: In situ analysis of CCR8+ regulatory T cells in lung cancer: suppression of GzmB+ CD8+ T cells and prognostic marker implications
Source: BMC Cancer. 2024 May 23;24:627. doi: 10.1186/s12885-024-12363-x (PMC11112935; doi:10.1186/s12885-024-12363-x)
Supplement: Supplementary file 3 — Supplementary Material 3. [file 12885_2024_12363_MOESM3_ESM.pdf]

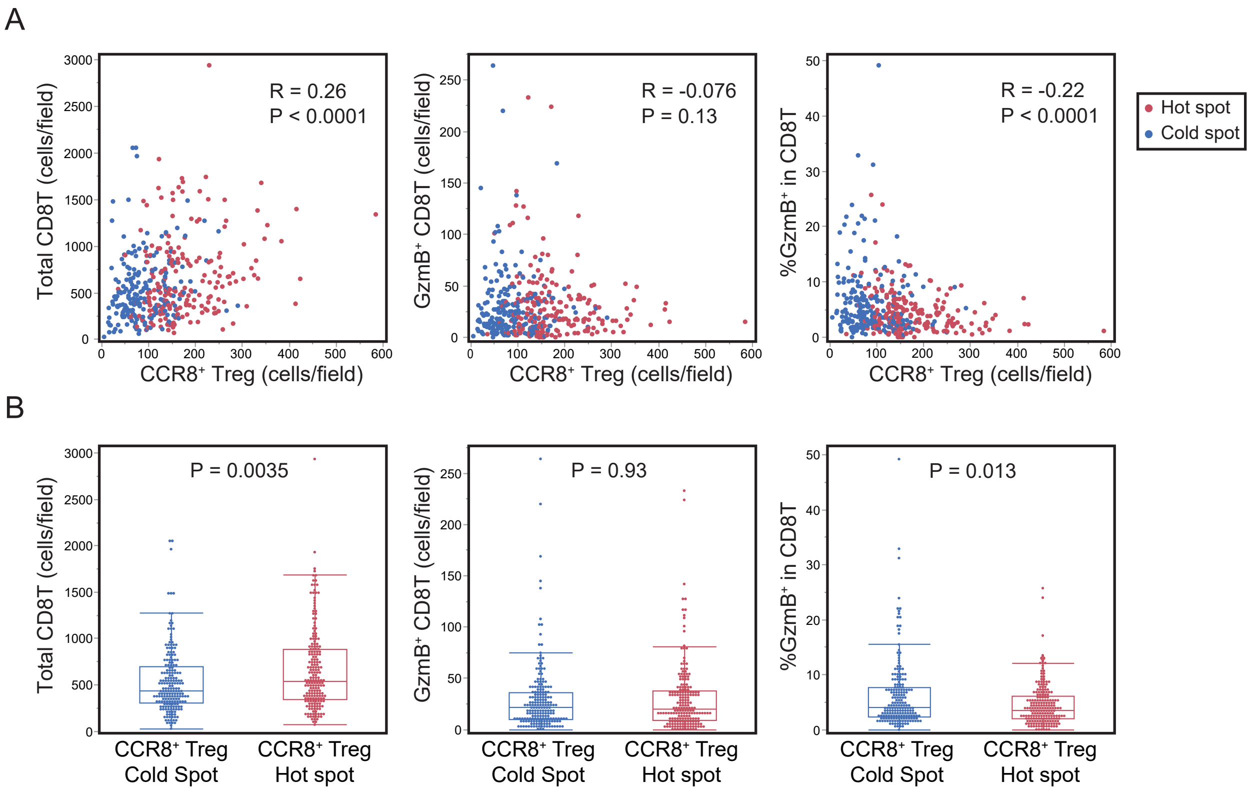

Supplementary Figure S3. Association of $CCR8^+$ Tregs with $CD8^+$ T cell parameters in Hot and Cold Spots. Both the Hot Spots and Cold Spots (five fields per case) were analyzed for the top 41 lung squamous cell carcinoma (LSCC) patients with high $CCR8^+$ Treg infiltration in Hot Spots. **A.** The correlation plots with the linear regression model of the data from 410 fields are displayed. **B.** Each $CD8^+$ T cell parameter in the Hot Spots and Cold Spots is shown in boxplots and compared by the Mann-Whitney U test.
