# Supplementary material for: In situ analysis of CCR8+ regulatory T cells in lung cancer: suppression of GzmB+ CD8+ T cells and prognostic marker implications
Source: BMC Cancer. 2024 May 23;24:627. doi: 10.1186/s12885-024-12363-x (PMC11112935; doi:10.1186/s12885-024-12363-x)
Supplement: Supplementary file 4 — Supplementary Material 4. [file 12885_2024_12363_MOESM4_ESM.pdf]

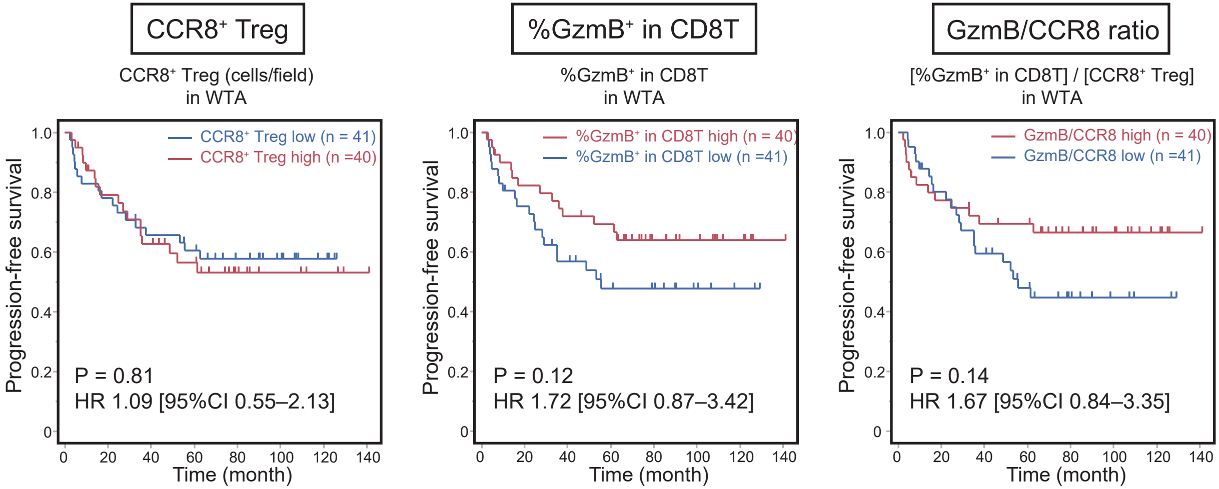

Supplementary Figure S4. Association of Treg and $CD8^+$ T cell profiles by the whole tumor area (WTA) analysis protocol with prognosis. The 81 lung squamous cell carcinoma (LSCC) patients were divided into high and low groups relative to the median value of each measurement by the WTA analysis. The Kaplan-Meier survival curves for progression-free survival (PFS) are presented. Group comparisons were conducted using the log-rank test. The hazard ratios (HRs) with the 95% confidence interval (CI) were calculated using the Cox proportional hazards model.
