# Supplementary material for: In situ analysis of CCR8+ regulatory T cells in lung cancer: suppression of GzmB+ CD8+ T cells and prognostic marker implications
Source: BMC Cancer. 2024 May 23;24:627. doi: 10.1186/s12885-024-12363-x (PMC11112935; doi:10.1186/s12885-024-12363-x)
Supplement: Supplementary file 5 — Supplementary Material 5. [file 12885_2024_12363_MOESM5_ESM.pdf]

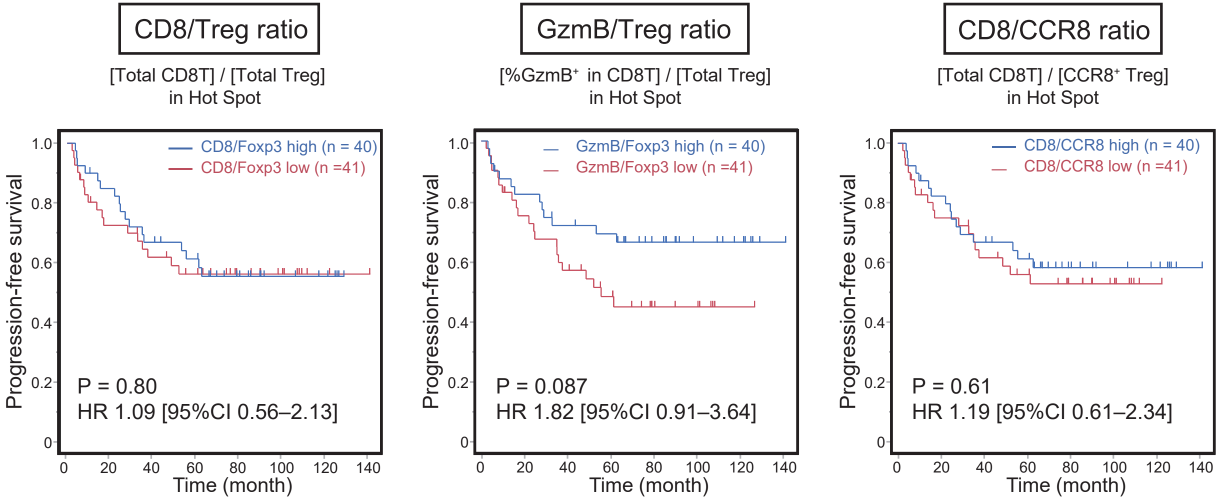

Supplementary Figure S5. Association of various Treg and $CD8^+$ T cell balance indicators with prognosis. Using the average measurements obtained from the five Hot Spots per case by the region of interest (ROI) analysis protocol, 81 lung squamous cell carcinoma (LSCC) patients were divided into high and low groups relative to the median value of each measurement. The Kaplan-Meier survival curves for progression-free survival (PFS) are presented. Group comparisons were conducted using the log-rank test. The hazard ratios (HRs) with the 95% confidence interval (CI) were calculated using the Cox proportional hazards model.
